# Acquisition of Apoptotic Resistance in Cadmium-Transformed Human Prostate Epithelial Cells: Bcl-2 Overexpression Blocks the Activation of JNK Signal Transduction Pathway

**DOI:** 10.1289/ehp.10075

**Published:** 2007-04-05

**Authors:** Wei Qu, Hengning Ke, Jingbo Pi, Daniel Broderick, John E. French, Mukta M. Webber, Michael P. Waalkes

**Affiliations:** 1 Inorganic Carcinogenesis Section, Laboratory of Comparative Carcinogenesis, National Cancer Institute at the National Institute of Environmental Health Sciences and; 2 Laboratory of Molecular Toxicology, National Institute of Environmental Health Sciences, National Institutes of Health, Department of Health and Human Services, Research Triangle Park, North Carolina, USA; 3 Departments of Zoology and Medicine, Michigan State University, East Lansing, Michigan, USA

**Keywords:** apoptosis, Bcl-2, cadmium, JNK, malignant transformation

## Abstract

**Background:**

We have recently shown that cadmium can induce malignant transformation of the human prostate epithelial cell line (RWPE-1) and that these cadmium-transformed prostate epithelial (CTPE) cells acquire apoptotic resistance concurrently with malignant phenotype.

**Objective:**

The present study was designed to define the mechanism of acquired apoptotic resistance in CTPE cells.

**Methods:**

Various molecular events associated with apoptosis were assessed in control and CTPE cells that were obtained after 8 weeks of continuous cadmium exposure.

**Results:**

Compared with control, CTPE cells showed a generalized resistance to apoptosis induced by cadmium, cisplatin, or etoposide. Signal-regulated mitogen-activated protein kinases, extracellular signal-regulated kinases 1 and 2, c-Jun N-terminal kinases (JNK1 and JNK2), and p38 were phosphorylated in a cadmium concentration-dependent fashion in CTPE and control cells. However, phosphorylated JNK1/2 levels and JNK kinase activity were much lower in CTPE cells. The pro-apoptotic gene *Bax* showed lower transcript and protein levels, whereas the anti-apoptotic gene *Bcl-2* showed higher levels in CTPE cells. The ratio of *Bcl-2/Bax*, a key determinant in apoptotic commitment, increased more than 4-fold in CTPE cells. In *Bcl-2*–transfected PT-67 cells, phosphorylated JNK1/2 levels were much lower after apoptogenic stimulus, and apoptosis induced by cadmium or etoposide was reduced compared with control. Mutation of tyrosine to serine at the 21st amino acid of the Bcl-2 protein BH4 domain resulted in a loss both of suppression of JNK1/2 phosphorylation and its anti-apoptotic function.

**Conclusions:**

CTPE cells become resistant to apoptosis during malignant transformation, and disruption of the JNK pathway and Bcl-2 overexpression play important roles in this resistance. Bcl-2 BH4 domain is required for modulating JNK phosphorylation and anti-apoptotic function.

Prostate cancer is the second leading cause of cancer-related deaths in American men (Greenlee et al. 2001). Worldwide both the incidence and mortality rates for prostate cancer are recently increasing, even in low-risk populations (Hsing et al. 2000). However, the etiology of prostate cancer remains incompletely defined. Epidemiologic evidence indicates that cadmium, a known human carcinogen, is a potential prostatic carcinogen (International Agency for Cancer Research 1993; Waalkes 2003), and rodent studies clearly show cadmium can induce prostate cancer (Waalkes 2003). In addition, *in vitro* model systems show cadmium can induce malignant transformation of both human (Achanzar et al. 2001) and rodent prostate epithelial cells (Terracio and Nachtigal 1988). Indeed, our recent work showed that cadmium can induce malignant transformation of the human prostate epithelial cell line (RWPE-1) (Achanzar et al. 2001) and that these cadmium-transformed prostate epithelial (CTPE) cells acquire apoptotic resistance concurrently with malignant phenotype (Achanzar et al. 2002). However, the exact mechanism by which cadmium induces malignant transformation is unclear.

Apoptosis is a form of cell suicide that plays an important role in development and maintenance of tissue homeostasis in multicellular organisms (Shivapurkar et al. 2003). Apoptosis also plays an essential role in the elimination of mutated or transformed cells from the body (Shivapurkar et al. 2003). Disruption of apoptosis plays a major role in tumor formation and malignant progression (Lowe and Lin 2000). In fact, acquired resistance toward apoptosis is a hallmark of most types of cancer (Hanahan and Weinberg 2000). Tumor cells and their precursors have multiple mechanisms to escape or avoid apoptosis that favor survival. The expansion of a tumor cell population is determined not only by the rate of cell proliferation but also by the rate of cell apoptosis. Tumor growth thus depends on the balance between cell proliferation and apoptosis. Therefore, acquired resistance to apoptosis could have important implications in both tumor initiation and progression.

Mitogen-activated protein kinases (MAPKs) comprise a family of serine/threonine phosphorylating proteins that mediate the signal transduction pathways from a variety of extracellular signals to regulate the expression of specific genes (Qu et al. 2002; Xia et al. 1995). The extracellular signal-regulated kinases (ERKs) typically transduce growth factor signals that cause cell differentiation or proliferation. Stress signals activate the c-Jun NH_2_-terminal kinase (JNK) and p38 path-ways to produce stress response, growth arrest, and apoptosis. These MAPKs are activated by the dual phosphorylation of specific tyrosine and threonine residues, which is performed by regulatory kinases upstream in the signaling cascade. Although activation of JNK and p38 MAPK pathways has been associated with induction of apoptosis (Xia et al. 1995), the precise mechanisms involved in the JNK-induced apoptotic response remain to be determined.

Three subfamilies of Bcl-2 [Gene ID: 24224; National Center for Biotechnology Information (NCBI) 2007) proteins have been identified to play important roles in the apoptotic response. Of these subfamily members, the Bcl-2 subfamily functions to inhibit apoptosis, whereas the Bax (Gene ID: 12028; NCBI 2007) and BH3 subfamilies tend to promote apoptosis (Gross et al. 1999). Bcl-2 is an intracellular membrane-associated protein that can prevent cell death induced by a variety of apoptotic stimuli (Park et al. 1997). It has been demonstrated that the Bax subfamily is essential for apoptotic signal trans-duction by JNK (Frisch et al. 1996; Park et al. 1996, 1997). The JNK signaling pathway is required for stress-induced release of mitochondrial cytochrome *c* and apoptosis (Lei et al. 2002).

The purpose of the present study was to define the mechanisms of apoptotic resistance and any concurrent alterations in signal trans-duction pathways in cadmium-induced malignant transformation using our established *in vitro* human prostate epithelial carcinogenesis cell model system. Using this system, we found that cadmium-transformed cells acquired tolerance to acute cadmium cytotoxicity and became highly resistant to chemically induced apoptosis. This acquired apoptotic resistance in cadmium-transformed cells appeared to be due to overexpression of Bcl-2, which blocked the activation of the JNK signaling pathway by various apoptotic stresses.

## Materials and Methods

### Chemicals and reagents

Cadmium chloride (CdCl_2_), etoposide, cisplatin, chloroform, isopropanol, formaldehyde, and ethidium bromide were purchased from Sigma Chemical Co. (St. Louis, MO). A nonradioactive cell proliferation assay kit was obtained from Promega (Madison, WI).

### Cell line and treatment

The development of the human nontumorigenic parental RWPE-1 cell line, used as the control in this work, and the derivation of the cadmium-transformed prostate epithelial cell line are described in detail elsewhere (Achanzar et al. 2001; Bello et al. 1997; Webber et al. 2001). Although immortalized, RWPE-1 cells have retained properties exhibited by normal prostate epithelium *in vivo*. The CTPE cell line was developed by chronic cadmium exposure of the RWPE-1 cell line as previously described, and CTPE cells form aggressive, prostate carcinoma-like tumors upon inoculation into nude mice (Achanzar et al. 2001). The observed malignant transformation in CTPE cells is not reversible after removal from cadmium. To define steady-state events and to prevent acute phase effects of cadmium, cells were placed in cadmium-free media for 2 or more weeks before the assessment of experiments. Control and CTPE cells were grown in keratinocyte serum-free medium (KSFM) containing 50 μg/mL bovine pituitary extract (BPE), 5 ng/mL epidermal growth factor (EGF), and 1 × antibiotic/antimycotic mixture (10,000 U/mL penicillin G sodium, 10,000 μg/mL streptomycin sulfate, 25 μg/mL amphotericin B). Cultures were incubated at 37°C in a humidified atmosphere containing 5% CO_2_. Cells were passed weekly.

### Establishing Bcl-2 producing cell line

Murine *Bcl-2* from pUSEamp-Bcl2 (Upstate Biotechnologies, Lake Placid, NY) was sub-cloned into pLNCX2 (OriGene Technologies, Rockville, MD) as an 816-bp HindIII-XbaI fragment. Bcl-2 expression was under the control of the cytomegalovirus promoter. A Quick-change site-directed mutagenesis kit (Stratagene, La Jolla, CA) was used to replace the A nucleotide with a C in the coding sequence, leading to the substitution of a serine for a tyrosine at the 21st amino acid in the protein sequence. Mutagenesis forward and reverse primers used were 5′-ATGAAGTACATA CATTCTAAGCTGTCACAGAGG-3 and 5′-TCTGTGACAGCTTAGAATGTATG TACTTCATC-3′, respectively. The sequence-verified wild-type *Bcl-2*, mutated *Bcl-2,* and pLNCX2 empty vector plasmid were trans-fected separately into the BD RetroPACK PT-67 (NIH 3T3) cell line. After 10–14 days of growth (G418 selection, 750 μg/mL), large visible clones were isolated and transferred to individual dishes (Huang et al. 1997). Clones with high expression of Bcl-2 protein or mutant Bcl-2, termed PT67-Bcl-2 and PT67-Bcl2Y21S, were maintained for further analysis. The level of the endogenous Bcl-2 in PT67-Vector was too low to be detected. All cell lines were cultured in Dulbecco’s modified Eagle’s media containing 10% fetal bovine serum, 100 μg/mL streptomycin sulfate, and 100 U/mL penicillin G sodium.

### Cell proliferation

We used the Promega Cell Titer 96 Non-Radioactive Cell Proliferation Assay kit (Promega) to determine acute cytotoxicity of cadmium. This assay measures the amount of formazan produced by metabolic conversion of Owen’s reagent [3-(4,5-dimethylthiazol-2-yl)-5-(3-carboxymethoxyphenyl)-2-(4-sulfophenyl)-2*H*-tetrazolium, inner salt, MTS] by dehydrogenase enzymes found in the mitochondria of metabolically active cells. The quantity of formazan product, as measured by absorbance at 490 nm, is directly proportional to the number of living cells. A minimum of four replicates of 10,000 cells per well were plated in 96-well plates and allowed to adhere to the plate for 24 hr, at which time the media was removed and replaced with fresh media containing the cadmium. Cells were then incubated for an additional 24 hr and cell viability was determined (Qu et al. 2001, 2005). We determined the LC_50_ (median lethal concentration) values by analyzing the linear portion of the metabolic integrity curves and comparing between different cells.

### Determination of apoptosis by flow cytometry

Detection of phosphatidylserine on the outer plasma membrane of apoptotic cells was performed using Annexin V and propidium iodide according to the manufacturer’s recommendations (Trevigen, Inc. Gaithersburg, MD). For each sample, 10,000 cells were examined by flow cytometry using a Becton Dickinson FACSort (Becton Dickinson, Franklin Lakes, NJ). The percent of apoptotic cells was determined by analysis of the dot plots using CellQuest software (Qu et al. 2002).

### Western blot analysis

After cells were grown to 70–80% confluence, they were placed in supplement-free medium for 24 hr. Cells were then washed with ice-cold phosphate-buffered saline, and scraped into cell lysis buffer containing 50 mM HEPES (pH 7.5), 150 mM NaCl, 1.5 mM MgCl_2_, 50 mM pyrophosphate, 1 mM sodium orthovanadate, 1 mM EGTA, 100 mM sodium fluoride, 1% Triton X-100, 10% glycerol, 10 μg/mL leupeptin, 10 μg/mL aprotinin, and 1 mM phenylmethylsulfonyl fluoride. The cells were incubated in lysis buffer for 30 min on ice, sonicated, and centrifuged at 14,000 × *g* for 15 min. The supernatant was designated as the cell lysate (Qu et al. 2002). Protein concentration was determined by the Bio-Rad method (Bio-Rad, Hercules, CA). Protein samples (20 μg) derived from the various cell preparations were subjected to sodium dodecyl sulfate–polyacrylamide gel electrophoresis and transferred onto nitrocellulose membranes. The membranes were blocked with 5% nonfat dry milk in Tris-buffered saline containing 0.05% Tween 20 and probed with various antibodies. After incubation with secondary antibodies, immunoblots were visualized with the LumiGlo detection method purchased from New England Biolabs (Beverly, MA).

### JNK activity assay

We determined the enzymatic activity of JNKs using commercially available kits (New England Biolabs, Beverly, MA). Briefly, cell extracts containing 250 μg total protein were incubated overnight at 4°C with an N-terminal c-Jun fusion protein bound to glutathione–Sepharose beads for JNK kinase activity assay. The kinase reaction was performed by adding 100 μmol/L adenosine triphosphate to the suspension. Phosphorylation of c-Jun was measured by Western blot analysis with a phospho-specific c-Jun antibody that specifically detects Ser63-phosphorylated c-Jun, a site important for c-Jun-dependent transcriptional activity (Qu et al. 2002).

### Quantitative real-time RT-PCR analysis

Total RNA was isolated using TRIzol (GIBCO/BRL Life Technologies, Bethesda, MD), then subjected to DNase digestion by using a RNase-Free DNase set (Qiagen, Valencia, CA) followed by the cleanup using a RNeasy Mini kit (Qiagen). The resultant DNA-free RNA was quantitated by ultraviolet spectroscopy at 260 nm and stored in RNase-free H_2_O at –70°C. Quantitative real-time RT-PCR was conducted as described previously (Waalkes et al. 2004). Briefly, total RNA from each sample was reverse transcribed with murine leukemia virus (MuLV) reverse transcriptase (Applied Biosystems, Foster City, CA) and oligo-d(T) primers. The SYBR Green PCR Kit (Applied Biosystems) was used for quantitative real-time RT-PCR analysis. The primers were designed using Primer Express software (Applied Biosystems) and are listed here: Bax, Forward CATGGAGCT GCAGAGGATGAT; Reverse GTCAGCT GCCACTCGGAAAA. Bcl-2, Forward TC CCTCGCTGCACAAATACTC; Reverse TTCTGCCCCTGCCAAATCT. β-actin, Forward ACTGGAACGGTGAAGGTGA CA; Reverse ATGGCAAGGGACTTCCTG TAAC. Relative differences in gene expression between groups were expressed using cycle time (Ct) values. These values were first normalized with that of β*-actin* in the same sample, and the expression in the experiment group was expressed as a percentage of expression in controls. Real-time fluorescence detection was carried out using a MyiQ singleColor Real-Time PCR Detection System (Bio-Rad).

### Statistical analysis

For data expressed as mean ± SE, Student’s *t*-test or analysis of variance with subsequent Dunnett’s test was used as appropriate. Incidence data were tested by Fisher’s exact test. Values are derived from three or more replicates. Differences were considered significant at *p* < 0.05.

## Results

### Acute cytotoxicity of cadmium

Continuous cadmium exposure for 8 or more weeks induces malignant transformation in RWPE-1 cells, producing the tumor-forming CTPE transformant (Achanzar et al. 2001). In the present study, CTPE and passage-matched RWPE-1 control cells were treated with cadmium for 24 hr, and cytotoxicity was measured. CTPE cells clearly acquired tolerance to the acute toxic effects of cadmium. The LC_50_ value for CTPE cells exposed to cadmium was 15.5 ± 1.9 μM compared with 7.0 ± 0.5 μM in control cells—a 2.2-fold difference reflecting a marked reduction in sensitivity.

### CTPE cells acquire generalized resistance to chemically induced apoptosis

**Figure 1:**
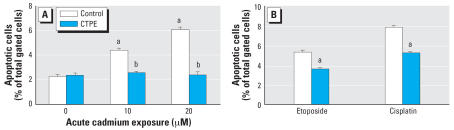
CTPE cells acquire generalized resistance to apoptosis. (*A*) CTPE and control RWPE-1 cells were treated with 10 or 20 μM cadmium for 24 hr. (*B*) CTPE and control cells were treated with etoposide (50 μg/mL) or cisplatin (10 μM) for 24 hr. Apoptosis was determined by flow cytometry using fluorescent-labeled annexin V and propidium iodide. Data represent the percent of apoptotic cells in lower right quadrants. Results are presented as the mean ± SE, *n* = 3. ^***a***^Significantly (*p* < 0.05) different from cell-line–matched untreated cells; ^***b***^significantly (*p* < 0.05) different from dosage-matched control cells.

To determine if acquired apoptotic resistance occurred concurrently with cadmium-induced malignant transformation, control and CTPE cells were treated with cadmium and apoptosis was determined by flow cytometry. Cadmium-induced apoptosis was markedly reduced in CTPE cells compared with control cells (Figure 1A). These results indicate that after transformation has occurred, cells acquired a marked resistance to apoptosis induced by cadmium. Furthermore, to detect whether the CTPE cells possessed generalized apoptotic resistance, control and CTPE cells were treated with etoposide, a DNA repair inhibitor and potent apoptogen, or cisplatin, a chemo-therapeutic compound, for 24 hr. Analysis of the flow cytometry data revealed that CTPE cells had about 40% fewer apoptotic cells than control when exposed to either etoposide or cisplatin (Figure 1B).

### Cadmium-transformed phenotype shows reduced JNK1/2 phosphorylation and JNK1/2 kinase activity

**Figure 2:**
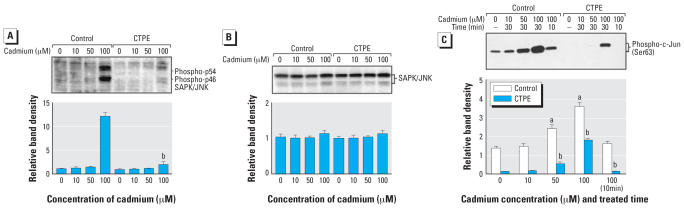
Effect of acute cadmium treatment on levels of phosphorylated JNK1/2 and JNK1/2 kinase activity in CTPE and control cells. Cells were acutely exposed to various concentrations of cadmium for 30 min. (*A*) The levels of phosphorylated JNK1/2 were determined by Western blot analysis (upper panel) and were analyzed by scanning densitometry (lower panel). (*B*) After development, the membrane was stripped and reprobed with regular antibodies against JNK1/2 (upper panel) and was analyzed by scanning densitometry (lower panel). (*C*) Cells were treated with cadmium for 30 min at the levels indicated for the concentration response assessment or were treated with 100 μM cadmium for the time course assessment. Cell extracts were incubated overnight with c-Jun fusion protein. Phosphorylation of c-Jun at Ser263 was measured by Western blot using phospho-c-Jun (Ser263) antibody. Results were analyzed by scanning densitometry. Blot represents a typical result of three independent experiments. The values were standardized to untreated control as 1. Results are presented as the mean ± SE; *n* = 3 experiments. ^***a***^Significantly (*p* < 0.05) different from cell-line matched untreated cells; ^***b***^significantly (*p* < 0.05) different from dosage-matched control cells.

The JNK and p38 pathways have been implicated in the regulation of apoptosis induced by various stimuli (Xia et al. 1995). Several recent articles have suggested that ERK might also be involved in apoptotic signaling (e.g., Mohr et al. 1998). To determine which signal pathway may be involved in cadmium-induced apoptosis, cells were acutely subjected to cadmium (0, 10, 50 or 100 μM) for 30 min. The levels of phosphorylated ERK1/2, JNK1/2, and p38 were determined by Western blot analysis. All three MAPKs were phosphorylated in a cadmium concentration-dependent fashion both in control and CTPE cells. Interestingly, the level of phosphorylated JNK1/2 was markedly decreased in the CTPE cells compared with control cells (Figure 2A). The membrane used to define the phosphorylated form was stripped, then reprobed with antibody against JNK that recognizes both the phosphorylated and nonphosphorylated forms to assess total JNK1/2. As shown in Figure 2B, no major differences were observed in the levels of total JNK1/2. Thus, the decrease in phosphorylated JNK represents a decrease in phosphorylation of the native protein in transformed cells rather than a reduction in total cellular JNK protein. The levels of both phosphorylated ERK1/2 and p38 did not show any significant differences between control and CTPE cells (data not shown). Dual phosphorylation of JNKs at Thr183/Tyr185 is essential for kinase activity and phosphorylation at these sites is an excellent marker of JNK activity. To confirm JNK activation, an *in vitro* kinase assay was performed using a c-Jun N-terminal fusion protein as the substrate. After acute cadmium exposure, JNK kinase activation was markedly reduced in CTPE cells compared with control cells in both the concentration–response and time-course analysis (Figure 2C). The levels of phosphorylation of JNK determined by using the phospho-specific antibody were thus consistent with the kinase activity of JNK in cell extracts.

### Effect of Ro318220 on JNK1/2 kinase activity and apoptosis

**Figure 3:**
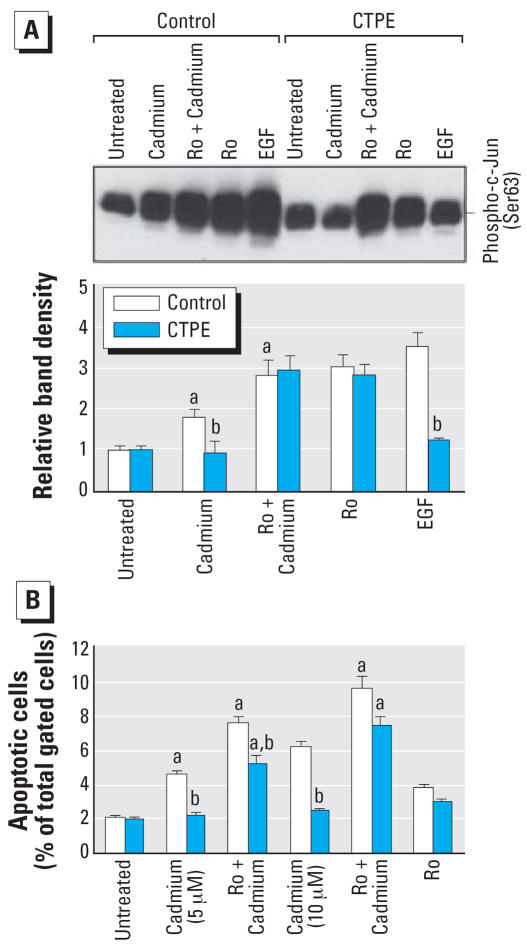
Effect of Ro318220 on cadmium-induced JNK1/2 kinase activity and apoptosis. (*A*) CTPE and control cells were pretreated with 10 μM Ro318220 (Ro), an activator of JNK, for 30 min, followed by cadmium (100 μM) treatment for 30 min. One group of each cell type was also treated with EGF (10 ng/mL) for 5 min. Activity of JNK1/2 kinase was then measured as described in Figure 2C (upper panel). Results were analyzed by scanning densitometry (lower panel). Blot represents one of three independent experiments. (*B*) CTPE and control cells were pretreated with 10 μM Ro318220 for 30 min, followed by cadmium (5 or 10 μM) treatment for 24 hr. Apoptosis was determined by flow cytometry. Data are given as the proportion (%) of the total number of cells gated that are apoptotic cells. Results are presented as the mean ± SE, *n* = 3 experiments. ^***a***^Significantly (*p* < 0.05) different from cell-line matched untreated cells; ^***b***^Significantly (*p* < 0.05) different from dosage-matched control cells.

The compound Ro318220 (Ro) is a strong activator of JNK (Beltman et al. 1996; Sandau et al. 1999). Thus, studies were designed to determine if Ro318220 could bypass the blockage of JNK kinase activity in CTPE cells. Cells were pre-treated with Ro318220 (10 μM, 30 min) followed by acute cadmium exposure (100 μM, 30 min), and an *in vitro* kinase assay was performed using a c-Jun N-terminal fusion protein as the substrate. As shown in Figure 3A, after cadmium treatment JNK kinase activity was much lower in CTPE cells compared with control, confirming early experiments. However, pretreatment with Ro318220 before cadmium effectively bypassed this block in JNK activity, bringing JNK kinase activity in CTPE cells to control levels. EGF, used as a positive control for stimulating JNK activity, was much less effective in CTPE than control cells. These results suggest that the activation of the JNK pathway is perturbed in CTPE cells, specifically at the point of JNK phosphorylation. To confirm that the effects of Ro318220 depend JNK1/2 activation and are not due to protein kinase C (PKC) inhibition, similar experiments were performed with staurosporine (40 nM) and GF109203 (2.5 mM), two well-known PKC inhibitors (Beltman et al. 1996; Sandau et al. 1999). In contrast to the effects of Ro318220 on JNK kinase activation, both staurosporine and GF109203 did not affect JNK kinase activation in either control or CTPE cells. To determine if JNK activation through Ro318220 could bypass the apoptotic block seen after cadmium-induced transformation, cells were pretreated with Ro318220 before cadmium treatment and apoptosis was assessed (Figure 3B). Cadmium was again markedly less effective in inducing apoptosis in CTPE cells compared with control. The addition of Ro318220 before acute cadmium treatment significantly increased apoptotic rate between 2.6- and 3.7-fold in CTPE cells.

### Expression of pro- and anti-apoptotic genes

**Figure 4:**
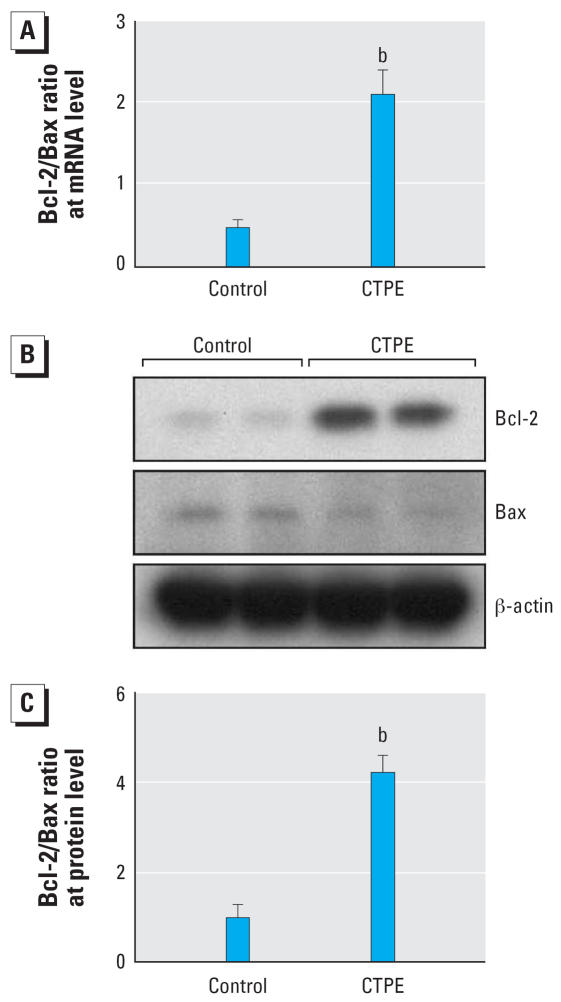
Effect of transformation on the ratio of Bcl-2/Bax at transcription and protein levels. (*A*) Real-time PCR of *Bcl-2* and *Bax* was performed in triplicate, the results were normalized to β*-actin* and are expressed as a ratio. (*B*) Western blot analysis of protein derived from CTPE and control cells using specific Bcl-2, Bax, and β-actin antibodies. Blot shown represents a typical result of four independent experiments. (*C*) The levels of Bcl-2, Bax, and β-actin protein were analyzed by scanning densitometry and used to calculate the ratio of Bcl-2/Bax based on β-actin. (*B*) indicates a significant (*p* < 0.05) difference from control cells.

Bax is an important pro-apoptotic protein and Bcl-2 is an important anti-apoptotic protein. It has been reported that multiple signal transduction pathways, including JNK, are capable of modifying Bcl-2 family members to reset susceptibility to apoptosis (Kharbanda et al. 2000; Yamamoto et al. 1999). Thus, the transcriptional levels of *Bcl-2* and *Bax* in CTPE and control were determined. Both *Bcl-2* and *Bax* transcript levels were significantly different in CTPE cells compared with control cells. The ratio of *Bcl-2/Bax* transcripts was increased over 2-fold in CTPE cells compared with control cells (Figure 4A). These results were confirmed by Western blot analysis (Figure 4B). The levels of Bcl-2 protein were substantially increased (~ 5-fold), whereas the level of Bax protein was markedly decreased (~ 2-fold) in the CTPE cells. The densitometric analysis showed that the ratio of Bcl-2/Bax, which is though to be an important determinant in dictating apoptosis, was significantly increased (more than 4-fold) in CTPE cells (Figure 4C), a finding indicative of reduced apoptosis.

### Bcl-2 suppresses the JNK1/2 phosphorylation and apoptosis induced by cadmium

**Figure 5:**
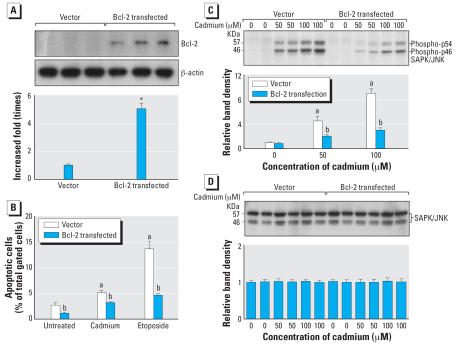
Immunoblot of Bcl-2 in cells expressing Bcl-2. BD RetroPACK PT67 cells were transfected with either a control vector or a vector containing *Bcl-2* as detailed in the “Materials and Methods” section. (*A*) Lysates were then immunoblotted with the Bcl-2 antibody (upper panel). After development, the membrane was stripped and reprobed with β-actin (middle panel). The levels of Bcl-2 and β-actin protein were analyzed by densitometry and used to calculate the increased fold based on β-actin (lower panel). Asterisk (*) indicates a significant (*p* < 0.05) difference between vector and *Bcl-2*–transfected cells. (*B*) Vector or *Bcl-2*–transfected cells were treated with cadmium (5 μM) or etoposide (50 μg/mL) for 24 hr. Apoptosis was determined by flow cytometry. ^***a***^Significantly (*p* < 0.05) different from untreated control; ^***b***^significantly (*p* < 0.05) different from dosage-matched control. (*C*) Vector or *Bcl-2*–transfected cells were exposed to cadmium for 30 min, the levels of phosphorylated JNK1/2 were determined by Western blot (upper panel) and analyzed by densitometry (lower panel). ^***a***^Significantly (*p* < 0.05) different from untreated control; ^***b***^significantly (*p* < 0.05) different from dosage-matched control. (*D*) After development, the membrane was stripped and reprobed with regular antibodies against JNK1/2 (upper panel) and analyzed by densitometry (lower panel). The values were standardized to untreated control as 1. Results are presented as the mean ± SE, *n* = 3 experiments.

To help define any possible interplay between Bcl-2 and JNK1/2, *Bcl-2* was transfected via a murine expression vector into PT-67 cells. Expression of Bcl-2 protein was detected stably in *Bcl-2*–transfected cells, but not in control cells (Figure 5A). Next, vector control and *Bcl-2*–transfected cells were treated with cadmium (5 μM) or etoposide (50 μg/mL) for 24 hr, and apoptosis was determined. Cadmium-induced apoptosis was markedly reduced in *Bcl-2*–transfected cells compared with vector control cells (Figure 5B). Moreover, etoposide-induced apoptosis was also significantly decreased in Bcl-2–expressing cells. Furthermore, the effect of acute cadmium treatment on JNK1/2 phosphorylation in either vector control or *Bcl-2*–trans-fected cells was examined. As expected, the level of phosphorylated JNK1/2 was markedly decreased in *Bcl-2*–transfected cells (Figure 5C). The membrane used to define the phosphorylated form was stripped, then reprobed with antibody against JNK that recognizes both the phosphorylated and non-phosphorylated forms to assess total JNK1/2. No major differences were observed in the levels of total JNK1/2 (Figure 5D). Taken together, these data suggest that Bcl-2 might modulate the JNK signaling cascade, which in turn prevents cadmium-induced apoptosis.

**Figure 6:**
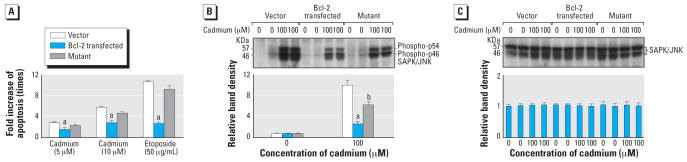
Mutant *Bcl-2* Y21 fails to protect against apoptosis. (*A*) Vector, *Bcl-2*–transfected, or mutant *Bcl-2* Y21 cells were treated with cadmium (5 or 10 μM) or etoposide (50 μg/mL) for 24 hr and apoptosis was determined by flow cytometry. Data are given as the fold increases of apoptosis based on untreated cells. (*B*) Vector, *Bcl-2*–transfected or mutant Bcl-2 Y21 cells were acutely exposed to cadmium (100 μM) for 60 min. The levels of phosphorylated JNK1/2 were determined by Western blot analysis (upper panel) and were analyzed by scanning densitometry (lower panel). (*C*) After development, the membrane was stripped and reprobed with regular antibodies against JNK1/2 (upper panel) and was analyzed by scanning densitometry (lower panel). Blot represents a typical result of three independent experiments. The values were standardized to untreated control as 1. Results are presented as the mean ± SE, *n* = 3 experiments. ^***a***^Significantly (*p* < 0.05) different from appropriate untreated control; ^***b***^significantly (*p* < 0.05) different from appropriate dosage-matched control.

To determine whether the BH4 region of Bcl-2 is essential for its anti-apoptotic function, Bcl-2 Y21 mutant cell line was established by site-specific mutagenesis, which replaced the tyrosine with a serine at the 21st amino acid of *Bcl-2* (Huang et al. 1997). Vector control, *Bcl-2*–transfected, and *Bcl-2* Y21 mutant cells were treated with cadmium or etoposide for 24 hr, and apoptosis was determined by flow cytometry. Both cadmium- and etoposide-induced apoptosis were markedly reduced in *Bcl-2*–transfected cells but restored largely in *Bcl-2* Y21 mutant cells (Figure 6A). Furthermore, the effect of acute cadmium treatment on JNK1/2 phosphorylation in vector control, *Bcl-2*–transfected, and *Bcl-2* Y21 mutant cells was examined. Most interestingly, the level of phosphorylated JNK1/2 was markedly decreased in *Bcl-2*–transfected cells but partially restored in *Bcl-2* Y21 mutant cells (Figure 6B). The membrane used to define the phosphorylated form was stripped, then reprobed with antibody against JNK that recognizes both the phosphorylated and nonphosphorylated forms to assess total JNK1/2. No major differences were observed in the levels of total JNK1/2 (Figure 6C). These data indicate that the Bcl-2 BH4 domain is involved in modulating JNK phosphorylation and its anti-apoptotic function.

## Discussion

It is unclear if cadmium acts as a carcinogen through genotoxic or epigenetic mechanisms. Whatever the basis, an important mechanism could be the disruption of signal transduction pathways resulting in aberrant cell accumulation. In this regard, the present study demonstrates that chronic cadmium-transformed prostate epithelial cells acquire generalized tolerance to chemically induced apoptosis. Disruption of apoptosis plays a major role in tumor formation and malignant progression. The activation of apoptosis is regulated by many different signals that originate from both intracellular and the extracellular sites (Vasilevskaya and O’Dwyer 2003). For example, the JNK signaling pathway has been implicated in the apoptotic response of cells exposed to stress. Several studies indicate that genotoxic stress induces translocation of stress-activated protein kinase (SAPK)/JNK to mitochondria (Kharbanda et al. 2000). Mitochondria are influenced by pro-apoptotic signal transduction through the JNK pathway, and the absence of JNK caused a defect in the mitochondrial death-signaling pathway, including the failure to release cytochrome *c* (Tournier et al. 2000). Therefore, JNK is required for stress-induced activation of the cyto-chrome *c*–mediated death pathway (Tournier et al. 2000). The results of the present study clearly demonstrate that JNK activation is perturbed in cadmium-transformed cells and that Ro318220, a strong activator of JNK, circumvents this apoptotic resistance. Therefore, cadmium-induced resistance to apoptosis appears to involve the specific suppression of the JNK pathways, which increases cell survival and decreases apoptosis (Vivo et al. 2003). In this regard, our prior work showed that after arsenite induced malignant transformation *in vitro*, generalized resistance to apoptosis develops, likely due to perturbation of the JNK pathway (Qu et al. 2002). In fact, these results are consistent with several reports that the stimulation of JNK is a prerequisite for cell apoptosis under various conditions, and a blockade of JNK activation results in the prevention of apoptosis (Verheij et al. 1996; Vivo et al. 2003; Xia et al. 1995). This blockade of apoptosis could be a key factor in cadmium carcinogenesis.

There are many possible targets of the JNK signaling pathway that may affect the mitochondria, including members of the Bcl-2 group of apoptotic regulatory proteins (Tournier et al. 2000). It has been reported that the anti-apoptotic protein Bcl-2 is phosphorylated and inactivated by JNK (Yamamoto et al. 1999). Bcl-2 disrupts a signaling cascade to the c-Jun N-terminal kinase activation induced by the apoptotic stresses (Park et al. 1997). However, the exact molecular mechanisms by which Bcl-2 prevents cell death remain unknown. It has been proposed that Bcl-2 might suppress cell death by modulating intracellular signaling cascades associated with apoptosis (Park et al. 1997). The cloning and characterization of the Bcl-2 oncogene established the importance of apoptosis in tumor development (Lowe and Lin 2000). For example, Bcl-2 promotes cell survival by blocking programmed cell death and disrupting normal proliferation (Hockenbery et al. 1990; McDonnell et al. 1989). It is recognized that Bcl-2 is expressed in a variety of human tumors. In particular, Bcl-2 overexpression is associated with prostate carcinoma (Herrmann et al. 1997). Conversely, Bax is a death promoter that is inactivated in certain types of cancer and plays critical roles in the initiation and execution of the apoptotic program (Putcha et al. 2003). The present study demonstrates that Bax protein levels were greatly reduced, and anti-apoptotic protein Bcl-2 expression markedly increased in CTPE cells as compared with RWPE-1 cells. *Bcl-2:Bax* ratio was approximately four times higher in CTPE cells than in control cells, which is consistent with perturbed apoptosis. The suppression of cell death could favor the development and progression of neoplasms by providing a selective survival advantage for transformed cells. Furthermore, upon investigation of a possible interplay between Bcl-2 and JNK1/2, we found the level of phosphorylated JNK1/2 was markedly decreased in a cadmium dose-dependent manner in *Bcl-2*–transfected cells. Also, cadmium- or etoposide-induced apoptosis was markedly reduced in Bcl-2–expressing cells compared with vector control cells. These data indicate that the intracellular signaling pathway for JNK stimulation is defective in Bcl-2–overexpressing cells, and Bcl-2 prevents both JNK signaling and cell death induced by various apoptotic stresses. The JNK signaling pathway might be downstream from the target of Bcl-2 action. Further study will be required to define precisely the interplay between JNK and Bcl-2.

The anti-apoptotic members of the Bcl-2 family contain four homology domains, BH1, BH2, BH3, and BH4 (Janumyan et al. 2003). The five most closely related mammalian homologues are Bcl-2, Bcl-x_L_ (Gene ID: 12048), Bcl-w (Gene ID: 12050), and Bax and Bak (Gene ID: 12018) (NCBI 2007). However, only the first three contain the N-terminal BH4 domain and inhibit apoptosis (Huang et al. 1998). BH1 and BH2 of Bax are critical for promoting cell survival, but Bax does not contain a region functionally equivalent to BH4 of Bcl-2 (Huang et al. 1998). To determine whether the BH4 region of Bcl-2 is essential for its anti-apoptotic function and regulation with JNK signal transduction pathway, we established a Bcl-2 Y21 mutant cell line by site-specific mutagenesis, which replaces the tyrosine with a serine at the 21st amino acid. Our results showed that *Bcl-2*–transfected cells were resistant to cadmium- or etoposide-induced apoptosis. Mutation of tyrosine at the 21st amino acid to serine resulted in loss of anti-apoptotic function that resulted in Bcl-2 Y21 mutant cells becoming as sensitive as vector control cells to apoptosis. Thus, the N-terminal BH4 domain is essential for the anti-apoptotic activity of Bcl-2, confirming previous published reports (Huang et al. 1998; Hunter et al. 1996). Most interestingly, our study showed that the level of phosphorylated JNK1/2 was markedly lower in *Bcl-2*–trans-fected cells than in vector control cells with cadmium treatment, but partially restored in *Bcl-2* Y21 mutant cells. Taken together, these data suggest that the Bcl-2 BH4 domain is required for modulating JNK phosphorylation and anti-apoptotic function.

In summary, after cadmium-induced malignant transformation has occurred in human prostate epithelial cells, transformed cells acquire resistance to apoptosis. The acquisition of apoptotic resistance appears to be linked to an increase in the anti-apoptotic action of Bcl-2 that perturbs the JNK signal transduction pathway. The Bcl-2 BH4 domain is required for modulating JNK phosphorylation and anti-apoptotic function. This acquired self-resistance to apoptosis may play an important role in cadmium-induced tumor initiation and progression.
